# Communication networks and team performance: selecting members to network positions

**DOI:** 10.3389/fpsyg.2023.1141571

**Published:** 2023-08-23

**Authors:** Jerry Guo, Linda Argote, Jonathan Kush, Jisoo Park

**Affiliations:** ^1^Frankfurt School of Finance and Management, Frankfurt, Germany; ^2^Tepper School of Business, Carnegie Mellon University, Pittsburgh, PA, United States; ^3^Charlton College of Business, University of Massachusetts Dartmouth, Dartmouth, MA, United States; ^4^School of Management, Clark University, Worcester, MA, United States

**Keywords:** communication networks, centrality, network positions, expertise, group performance

## Abstract

This study examines how individuals come to occupy communication network positions and the effect of selection processes on group performance. Drawing on the Carnegie perspective and research on communication networks, we compare the performance of groups whose members receive their choice of who occupies which network position to the performance of groups whose members do not receive their choice. We integrate ideas from the Carnegie perspective with the social psychological literature on the recognition of expertise to theorize that when group members choose who occupies which network positions, individuals select themselves and others into network positions that best suit their skillsets. The selection process allows groups to match individual member expertise to network position, thereby improving performance. We test this hypothesis in a laboratory study manipulating how members are assigned to positions in a centralized communication network. We find individuals who communicate more during training are more likely to be chosen as the central member, and that their communication activity explains the effect of choosing the central member on performance. Supplemental analyses suggest that groups allowed to select their central member performed as well as, and often better than, groups whose central member was randomly assigned. Our results contribute to the Carnegie perspective by demonstrating that the intra-team processes that develop a team’s network help explain their performance.

## 1. Introduction

In the Carnegie research perspective, the limits of individuals as information processors lead organizations to divide their goals into smaller units and develop structures that deal with these subgoals (March and Simon, 1958; Cyert and March, 1963). Communication networks structure the distribution of information and provide inputs for those who make key organizational decisions. The information that flows through communication structures in organizations is crucial for decision making (Cyert and March, 1963). Thus, organizations often rely on their communication networks to manage information (Cross and Prusak, 2002). Consequently, the nature of the communication networks and the individuals who comprise them have major implications for many organizational outcomes.

The Carnegie perspective studied the ways in which organizations gain efficiency through learning, routine development, and knowledge exchanges. Communication networks are a structure through which knowledge exchanges occur. Herbert Simon recognized that network experiments offer an ideal scenario to observe the ways in which ideas are adopted and to model cognitive limitations of communication capacity (Guetzkow and Simon, 1955). Early network research (see Shaw, 1964 for a review) focused primarily on the ways in which different network structures affect performance. A common structural characteristic of interest is a network’s centralization, the extent to which ties within the network are concentrated. Networks with a higher concentration of ties are “centralized,” whereas networks with a more even distribution of ties are “decentralized” (Katz et al., 2004). Considerable research from social psychology and communications scholars has compared the effect of centralized versus decentralized networks on group performance (Shaw, 1964), as well as how communication networks of various forms affect performance in groups (Sparrowe et al., 2001) and organizations (Balkundi and Harrison, 2006).

This study examines decisions about the development of communication networks on group performance to determine whether the process by which individuals come to occupy network positions influences team performance. We focus on decisions about who should occupy the central position in a centralized communication network, complementary to recent work on the structural influences of networks on performance (see Argote et al., 2018). Centralization captures the extent to which communication ties are concentrated in only one or a few members (Freeman, 1978). Centralized, dyadic communication networks are prevalent in organizations. For example, consider a team that spans multiple levels of an organizational hierarchy. In such a team, it is unlikely that a member at the bottom of the hierarchy will communicate directly with a member at the top. It is likely that communication will be dyadic, such that the member in the middle of the hierarchy will mediate communication. Similarly, consider a team that spans subunits within an organization such as when an engineer interacts with a marketing representative in addition to team members within their own department. The role of the engineer in this case is to serve as the link between their department and another unit in the organization; communication would be dyadic in this case because of the members’ roles.

Central members in a network are often responsible for gathering and sharing information (Cyert and March, 1963, p. 108). The Carnegie perspective speaks to formal and informal communication structures in organizations (Simon, 1997) but is silent about the processes through which workers come to occupy network positions. We extend the Carnegie perspective by showing that the process through which individuals come to occupy network positions affects the performance of networks. We integrate research on the Carnegie perspective with the social psychological research on expertise recognition to examine the process of assigning group members to network positions, specifically, which individual skills affect network position assignment. Subsequently, we compare the performance of networks in which members receive their choice of a central member to those where they do not.

We argue that individuals who occupy central positions within centralized networks require specific skillsets for the group to realize its performance potential. For example, a coordinator who interacts with unconnected team members in two separate departments occupies a central position, collecting and distributing information from the two unconnected team members and facilitating the work of the team. When the central member possesses skills such as communication and task expertise, a group’s performance potential is enhanced. Communication skills are necessary for individuals occupying central positions because those individuals control the information flow within a group (Freeman, 1978) which is imperative for successful task completion (Mesmer-Magnus and Dechurch, 2009). Task expertise benefits central members in interpreting information received from team members and orchestrating the team’s task performance strategy. For instance, in the above example, a coordinator would need to possess the ability to effectively relay task-relevant information to two team members who are not connected to each other. Additionally, the coordinator should have sufficient task expertise to comprehend and rephrase the information received from the two different departments. Drawing on the expertise recognition literature (e.g., Littlepage et al., 1997; Bunderson, 2003; Bonner and Baumann, 2012), we theorize that members learn about one another’s relevant skills as they work together (Argote, 2013) and that this knowledge enables members to select those who have the requisite skills for particular positions.

We build on this research tradition by examining how the process through which individuals come to occupy network positions affects the performance of networks. We use the controlled environment of a network experiment to investigate how networking choices affect group performance. In this way, we contribute to an understanding of how the development of a network, not just its structure, influences performance.

## 2. Theory and hypotheses

Individuals in work groups use communication networks, defined by which members communicate with one another, to accomplish their tasks (Shaw, 1964). In many settings, network structures are imposed by an organization through design or communication rules (Cyert and March, 1963). Centralized networks—where one or a few members are connected to more members relative to their peers—are common. In a centralized network, central members control the flow of communication within the group (Shaw, 1964; Freeman, 1978) and thus can be more influential than members in other network positions. Individuals within a firm can each possess unique knowledge and skills (March, 1991), but their ability to leverage those skills to benefit the firm may depend on their position within the network. For example, a member with exceptional communication skills would most benefit the firm if that skill were recognized and the member were placed in a network position, such as a central position.

Communication networks are frequently treated as dyadic in nature, where members communicate one-to-one with each other. The examples we provide in the introduction are representative of broader patterns of dyadic communication through which network structures emerge. Despite the rising prevalence of electronic communication, dyadic communication persists in organizations for multiple reasons. Hierarchies and roles in organizations can create status dynamics that favor centralized communication. Lower-level employees may not feel comfortable communicating directly with senior employees, preferring to communicate through an intermediary. Senior employees may feel that it is not appropriate or efficient for them to communicate with many lower-level employees. The nature of tasks that teams perform could also lead to dyadic communication, whereby team members interact directly with those relevant to the task at hand and do not broadcast information that is not relevant to others.

More importantly, individual team members may choose to communicate dyadically to mitigate their cognitive burdens. The concept of network inertia, though traditionally applied at the organization level, provides valuable insight into why individuals, bound by cognitive constraints, may sometimes favor dyadic communication (Kim et al., 2006). Individuals might prefer dyadic communication over all-channel communication due to the difficulty of managing a large volume of information and complex social relationships. Moreover, consistent dyadic communication with certain counterparts can establish shared routines, values, and languages. This familiarity obviates the need to reinvent the wheel with each interaction. In essence, the cognitive limitations of individuals in networks lead members in teams to make deliberate decisions about when and with whom to communicate. By being selective about communication, individuals can reduce the likelihood of information overload (Savolainen, 2007) and focus attention on information and tasks relevant to their work.

Findings in the literature about the influence of centralization on group performance are somewhat inconsistent. Early network research suggests that decentralized teams—where ties are evenly distributed between members—perform better on complex tasks (Shaw, 1964). Several more recent studies have found that decentralized network structures perform better than centralized structures on complex tasks. For example, Borgatti and Cross (2003) find that teams in the field with high centralization perform worse than those without such a structure. Balkundi and Harrison (2006) similarly find in a meta-analysis of field data that teams with high network density—strongly correlated with being decentralized—perform better than teams with low network density. In contrast, Ehrlich and Cataldo (2014), studying software development teams in the field, found that communication network centrality was associated with improved performance. Recent simulation (Lazer and Friedman, 2016) and experimental findings (Mason and Watts, 2011) also suggest that teams with centralized communication networks perform better on complex tasks than teams with decentralized structures. Other recent experimental work has also shown that centralized structures, as opposed to decentralized structures, can more efficiently integrate new members and thus new information into teams, even when their work is complex, thereby improving performance (Argote et al., 2018). Additionally, recent laboratory evidence shows that purely centralized five-person networks are better able to develop shared language and consequently perform better in an abstract symbol naming task than decentralized groups (Burt and Reagans, 2022; Reagans, 2022).

These disparate results suggest that additional factors outside of task complexity affect the performance of networks. It is not always clear, however, in non-experimental studies whether the effects of a network structure are driven by the network’s structural properties, the processes through which the structure emerged, and/or the characteristics of the person(s) who occupy network positions (Park et al., 2020). Laboratory studies benefit from the imposition of network structures and the random assignment of individuals to structures, which enable the causal identification of the effects of the networks on performance. Most of the above studies with conflicting result were conducted in the field where teams had already formed. We suggest that a key factor that may help explain these inconsistent findings is the process by which individuals enter network positions.

In this study, we examine the extent to which allowing group members to select who occupies the central position in a centralized communication network affects the group’s performance. In doing so, we bridge structural perspectives from laboratory studies with emergent perspectives from both the field and the laboratory. Through this bridging, we draw on insights from both the Carnegie perspective and psychology to investigate the member selection process. Our focus on whether and how an organization can gain efficiencies through worker choices in network formation could help explain a micro-foundation of the emergence of larger organizational structures, such as those described in the Carnegie perspective.

### 2.1. Communication networks and network positions in the Carnegie perspective

The Carnegie perspective represents a research tradition that emerged in the 1950s and 1960s from the work of scholars housed at the Graduate School of Industrial Administration at the Carnegie Institute of Technology. Emphasizing a plausibly realistic analysis of decision making within organizations, Carnegie perspective scholars introduced concepts like bounded rationality (Simon, 1957), coalitions (March, 1962), and problemistic search (Cyert et al., 1958) to the study of organizations.

Communication networks, in Organizations (March and Simon, 1958), influence decision processes in organizations, especially for non-programmed tasks. Coordination can be preprogrammed with planned responses to stimuli for programmed tasks, whereas communication networks facilitate organizational adaptation to emergent events in non-programmed tasks. Consequently, in these non-programmed scenarios, the shape of an organization’s communication network is particularly important, as only locally available information can be applied to the problem (March and Simon, 1958, p. 190).

March and Simon (1958) describe two general hypotheses about the emergence of communication networks in organizations. First, the more efficient a communication channel between two parties, the more it will be used. Second, a communication channel will be self-reinforcing (March and Simon, 1958, p. 189), such that a communication tie will evolve beyond its original purpose and encompass other purposes. The shape of the network that emerges has consequences for organizational outcomes by determining the frequency with which organizational members come into contact with one another and the information to which organization members are exposed. Thus, the network is important for both access to information and its transmission in solving problems.

The Carnegie perspective studied the development of communication structures and the effects of those structures on group performance. Communication networks facilitate organizational communication and problem solving, but they reflect the cognitive capacity limitations of individuals. Absent capacity limitations, networks could be fully decentralized, with all individuals connected to all others. We suggest that the process by which individuals come to occupy positions in communication networks can help individuals overcome limitations in their cognitive capacities.

Important research conducted at the Massachusetts Institute of Technology (MIT) was built on an innovative experimental platform for studying small group communication networks. The research assessed the effects of various networks on group performance (e.g., see Leavitt, 1951). Guetzkow and Simon (1955) extended Leavitt’s (1951) research by giving team members time between each task trial to communicate about how to organize themselves. Thus, in addition to examining the effects of the communication networks, the researchers examined how the communication networks shaped the patterns of information exchange in the groups.

Guetzkow and Simon (1955) studied three communication networks—wheel, all-channel, and circle—that affected the difficulty groups had in organizing themselves. Groups in the wheel condition had the least difficulty because they did not need to solve the organizational problems of eliminating communication channels, establishing relays, or determining who decides the solution, whereas the circle groups had to solve all three organizational problems and therefore had the most difficulty. Groups in the all-channel condition had an intermediate level of difficulty. Consistent with the researchers’ predictions about the difficulty of the task, the wheel groups organized earliest and completed the task trials most quickly. The all-channel groups organized more slowly than the wheel groups but eventually performed as well as groups in the wheel condition. The circle groups did not reach the performance of groups in the other two conditions during the study’s 20 trials. The researchers concluded that the communication networks do not affect the performance of the groups directly but rather do so indirectly through their influence on the ability of groups to organize themselves.

What Carnegie perspective research did not investigate is the process by which individuals are selected to network positions (which is rarely randomly determined in the field) and how this selection process influences performance. Research in social psychology speaks to member selection to position. Through collaborative interaction, individuals learn who possesses which skills. We argue that when teams determine members’ network positions, the selection process enables them to select members to occupy network positions that fit their skillsets and thereby improve group performance. This selection process, we suggest, is the mechanism that allows communication networks to overcome individual capacity limitations by creating a match between the capacity of the individuals and the requirements of the positions.

### 2.2. The recognition of roles and expertise

Network positions differ in nature within a given communication network. Network analysts identify roles within a network by identifying who has similar patterns of connections (Hanneman and Riddle, 2005). A given individual could share the same pattern of communication ties with another individual. For example, if two employees each had only one communication tie to a manager, these two employees would be considered equivalent to each other; if two managers were tied to two employees and a superior, the managers would also be considered equivalent to each other. The employees and managers each occupy network positions similar to others in their same functional role but different from those with a different role. The employees would engage in communication behaviors similar to other employees but different from their managers. We contend that network positions require specific skills that vary depending on the position within the network and that there can be a match (or mismatch) between an individuals’ skills and the requirements for the position they occupy.

Because our focus is on the selection of individuals to network positions, we turn to the literature on the recognition of expertise. Specifically, research on expertise recognition indicates that groups effectively identify members’ expertise when they have access to information about each other’s relative competencies (Liang et al., 1995; Bonner et al., 2002). One method for acquiring this information is through working together (Littlepage et al., 1997). Through collaborative work, members learn who possesses which skills and develop a shared understanding of the tasks at hand. Based on this shared understanding, group members assess each member’s skills, identify the expert, and give more weight to the expert’s opinions when making group decisions (e.g., Bonner, 2004; Bonner and Baumann, 2012). There is substantial work finding that teams perform better on decision-making tasks if members can identify and defer to their expert members (Yetton and Bottger, 1982; Stasser and Titus, 1987; Littlepage et al., 1995, 1997; Bonner et al., 2002; Bonner, 2004; Ho and Wong, 2009; Bonner and Baumann, 2012). Consequently, this line of research suggests that groups can assign members to network positions that best suit their expertise, and that such assignment will improve group performance.

### 2.3. Network position selection

Given that network positions require specific skills of those who occupy them, we suggest that one reason that individuals come to occupy network positions is because they have signaled their expertise to others who then select them into a specific position. Because they are prevalent and foundational to other networks, we focus on centralized communication networks and theorize about individual decisions around who should occupy specific positions in that network.

Centralized communication networks in their most elementary form consist of one central member who connects two otherwise disconnected alters. This central member is the sole communicator for the two disconnected members. Any information or communication the non-central members receive comes from the central member, and any information the central member receives must come from one or both alters. Consequently, as the communication core of the team (Humphrey et al., 2009). the central member plays the most important role in coordinating the work of the team and in managing communication; without a central member communicating, no information would flow through the team. The central member’s attributes are therefore of outsized importance to the team’s success.

Bunderson’s (2003) status characteristics perspective, an important theoretical framework in the expertise recognition literature, posits that members are more likely to identify experts on the basis of status characteristics, which could be specific (task-relevant) or diffuse (social categories such as age or sex). Initially, group members tend to rely on diffuse status characteristics to identify experts. However, as groups work together and have more opportunities to learn about other members’ task-relevant expertise, members increasingly utilize specific status characteristics. Similarly, Bonner et al. (2007) find that groups rely on expert members when they hold task-relevant information that can be used to gauge each member’s relative task competencies, whereas groups rely on members with high levels of extroversion when they lack such information. Again, this study suggests that groups focus on the cues of members’ task-relevant expertise when members have worked together and thus have information to evaluate group members’ task expertise. Finally, Bonner and Baumann (2012) hint that groups working together can facilitate the development of a shared understanding of the task requirements, and that this shared understanding makes it easier for groups to judge other members’ expertise.

We argue that the evocation of specific, task-relevant expertise among members of a team influences both the process by which individuals are selected into network positions and subsequent team performance. We suggest two primary criteria upon which this central member might be judged. First, members can be judged based on communication activity. We define communication activity as the volume of communication sent by an individual. In a centralized network, the group is forced to rely on the central member to coordinate work, as peripheral members are disconnected from one another and unable to understand the scope of the group’s knowledge. The extent to which an individual is communicating actively signals to others that they are capable of effectively relaying information (March and Simon, 1958) and thereby coordinating the team’s work effectively.

Second, members can be judged on task expertise. Central members in the communication structure not only need to communicate to coordinate the work of the team, but must interpret knowledge from the disconnected team members and either transmit that knowledge to where it is needed or to apply it to the task themselves. A member signals her expertise through her contributions to the task and through communication to others. Other team members who are presented with a centralized communication network are more likely to select an individual with task expertise to occupy the central position, recognizing the necessity of the central member in transferring knowledge across the team. Consequently, a member possessing task expertise will more likely be selected to occupy a central position.

As group members work together, they recognize which member possesses the most suitable skills to be the central member, such as the communication aptitude necessary to relay important task information and the task expertise needed to orchestrate the work of the group. Thus, we hypothesize:

*Hypothesis 1a: Individuals with higher communication activity are more likely to be selected as the central member than individuals with less frequent communication activity.*

*Hypothesis 1b: Individuals with higher task expertise are more likely to be selected as the central member than individuals with less task expertise.*

Next, we theorize how allowing groups to choose their central member affects the group’s performance. Research on expertise recognition indicates that groups can improve their task performance by recognizing members’ expertise and utilizing the skill sets of expert members in solving tasks (Littlepage et al., 1997; Bonner and Baumann, 2012). However, teams may not be able to make use of the diversity of the knowledge available in the team, and diversity in knowledge may have a positive or negative impact on a team’s ability to communicate and coordinate (Martins and Sohn, 2022).

We suggest that the selection process that places a team member with appropriate skills into a central network position mitigates penalties related to knowledge diversity and communication. The central member plays a critical role in sharing information and ideas between team members (Freeman, 1978), meaning the individual occupying the central position plays an outsized role in the team’s success. Teams that are able to select which members occupy network positions benefit because they are more likely to match team member characteristics to the requirements of the network position. Such a match would allow team members to complete tasks for which they are best suited, which benefits team performance by eliminating duplication of work and reducing errors (Liang et al., 1995).

A central member who has demonstrated communication activity can assign sub-tasks and coordinate the work of the group, and furthermore, can identify important information possessed by fellow group members and communicate that information to others. We argue that teams will be more likely to choose a central member who has demonstrated communication activity in previous interactions, and that the communication skills of the central member will improve performance.

Similarly, a central member with task expertise may volunteer such knowledge to help explicate the task requirements so that members with less expertise can better understand them and thereby better guide a group’s task-performance strategies than a central member lacking task expertise. We argue that teams that can select central members are more likely to have individuals with higher task expertise occupying the central position than teams that cannot select central members and that this helps explain their superior team performance.

*Hypothesis 2: Groups that receive their choice of a central member perform better than groups in which the central member is assigned.*

*Hypothesis 3a: The central member’s communication activity mediates the relationship between choice of central member and performance.*

*Hypothesis 3b: The central member’s task expertise mediates the relationship between receiving choice of central member and performance.*

## 3. Methods

We conducted a laboratory experiment to test our hypotheses. We collected a sample of 41 three-person groups for a total of 123 individuals participating. The groups were collected from a participant pool sponsored by a Mid-Atlantic University. The mean age in the sample was 21 years, 63% of the sample was Asian/Pacific Islander, and 61% of the sample was male. We had two experimental conditions. In one condition, members received their choice of who occupied the central position; in the other condition, members did not receive their choice. Participants were randomly assigned to groups, and groups were randomly assigned to conditions. As expected from the random assignment to conditions, there were no differences between the two conditions in terms of demographic representation.

### 3.1. Procedure

After arriving in the laboratory, each individual group member was placed into a separate room equipped with a computer where they were introduced to the study and asked to watch a training video and read introductory materials. Each group member’s computer was connected to a terminal computer so that group members worked collaboratively and simultaneously on a single project. Group members could only communicate dyadically via instant-messenger accessible on their computers, and experimenters controlled the communication network through the messaging client, meaning all interaction between participants was computer mediated for the duration of the experiment.

Groups worked collaboratively on a complex, graphical programming task using a programming interface called App Inventor. Rather than traditional programming (i.e., writing actual lines of code), participants were asked to program an Android application by manipulating graphical modules. These modules, each with a specific function, are placed together like jigsaw puzzle pieces to add features to an application. Groups were provided a partially completed Android application and instructed to add a new set of features to complete the application. Participants were shown the development canvas and an emulator that presented the current status of the application they were developing. The emulator running this application updated in real-time in response to changes made by group members. To ensure group member interdependence, each member received unique information about the application features they needed to add, meaning members needed to work together to determine which specific modules to add, how to combine these modules, and what the module settings should be.

Groups were given a 15-min practice period during which group members worked together on the task in an all-connected dyadic communication network. In this network, each member could communicate with the other two dyadically (i.e., one-to-one), but there was no option for all three members to communicate as a group. This practice period gave group members the opportunity to learn about one another’s expertise.

### 3.2. Manipulation

**FIGURE 1:**
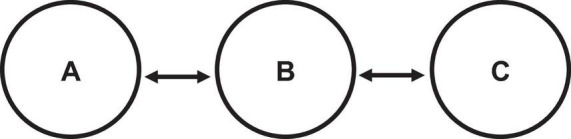
Centralized communication network. We provided the following instructions to participants to facilitate their understanding of the impending change in the communication structure and the role of the central member: “Your communication structure will change in the production task. So far, your team has communicated in an open communication structure. In the diagram below, the circles represent team members, and the lines represent communication links between them. Your communication structure for the production task will look like the diagram, with one member connecting two other members. How would you perform the task differently, and who would be the central member?”

Following the practice period, each participant completed an individual survey. Group members were then presented (as a group) with an image of a centralized communication network (Figure 1) where one member is the sole connector between two other disconnected members. In the centralized network, the two disconnected members cannot communicate directly with each other but can each communicate with the central member.

Group members were instructed to select each member to a network position and given 5 min to discuss via their all-connected dyadic network which group member should occupy each network position, as well as their strategy to complete the task in the centralized network. Then, as individuals, each group member identified which team member they wanted to occupy the central position in the network. We determined the group’s choice by identifying the member who received the most votes.

In the position chosen condition, the group was given their choice of central member. In the position assigned condition, we randomly selected one of the two non-selected members to occupy the central position. In both conditions, group members were able to make a choice, weakening the possibility that the choice process would explain any differences between the conditions. Eighteen groups received their choice of central member, and 23 groups did not receive their choice of central member. In both conditions, however, groups were told that their positions were randomly assigned. It was crucial for us to inform all groups that their assignment was random to reduce the possibility that teams in the position chosen condition would feel more motivated or perceive greater agency as they worked on the task. Our design enabled us to attribute any differences we found between conditions to having a member with the requisite characteristics occupy the central position rather than having a member the group believed they chose in the central position. We examine the potential trade-offs between the benefits and costs of this methodological choice more extensively in the discussion section.

Following the choice discussion and assignment, group members assumed their assigned network positions and were allotted 15 min to repeat the programming task in a production period where group performance was measured. Following their 15-min production period, group members completed a survey, were thanked and debriefed. We imputed means to address any missing data in the surveys.

### 3.3. Measures

#### 3.3.1. Performance

Performance was measured by the number of errors groups committed in adding features to the Android application. There was an objective standard, a correctly constructed program, against which each group’s output was compared.

#### 3.3.2. Communication activity

We measured communication activity by measuring the number of unique ideas sent by each group member during the training period prior to the selection of the central member. Communication in the task influenced performance by allowing the group to coordinate work and transfer information. We measured the volume of ideas an individual sent, as this was a visible indicator of a member’s ability to coordinate and convey information to fellow group members.

#### 3.3.3. App Inventor familiarity

We captured familiarity with App Inventor with a survey question on a 1–4 scale. Participants were asked, “How familiar are you with App Inventor?” Across the sample, the mean was 1.31, and the standard deviation was 0.62, suggesting that most participants had little prior familiarity with App Inventor. We constructed a variable capturing the relative difference between a focal member’s familiarity with App Inventor and the group’s average to capture how much a given member differed from their groupmates in terms of familiarity. We created this variable by averaging the App Inventor familiarity of the three team members. We then subtracted this average from each member’s reported familiarity. Higher values reflect more familiarity relative to the group’s average. We used this variable for our analysis because when members select their network positions, their point of reference is not absolute familiarity or skill, but a relative comparison with their fellow team members. This variable is our measure of task expertise as referred to in Hypotheses H1b and H3b.

### 3.4. Alternative explanation variables

We consider alternative explanations for Hypothesis 1 and Hypotheses 2 and 3. For Hypothesis 1, we identify additional reasons that an individual might be selected to occupy a central network position. Apart from demographic characteristics, prior research has found self-monitoring, or an individual’s ability to control their self-presentations, to be a predictor of occupying brokerage positions (Mehra et al., 2001) and individual centrality within a network (Sasovova et al., 2010; Fang et al., 2015). Additionally, we capture dominance motivation, whether individuals are naturally inclined to dominate in social situations, which could lead individuals to be selected for a central network position irrespective of their skills.

One alternative explanation for Hypotheses 2 and 3 is that individuals receiving their choice of a central member may feel a greater sense of control over their work and thereby be more highly motivated, performing better because they perceive control over their outcomes (Fisher, 1978; Spector, 1986). Informing all participants that positions were assigned randomly mitigated against a motivation effect. Additionally, evidence suggests that the opportunity to choose may not confer perceptions of control and thereby motivation (Klusowski et al., 2021). However, we account for the groups’ perceptions of control to investigate this alternative explanation.

#### 3.4.1. Self-monitoring

We measured individual self-monitoring with Lennox and Wolfe’s (1984) scale.

#### 3.4.2. Dominance motivation

We captured whether individuals are naturally inclined to dominate in social situations with the dominance motivation subscale of the achievement motivation scale (Cassidy and Lynn, 1989). This subscale captures a similar construct to social dominance orientation (Pratto et al., 1994) but is focused at the group level, whereas social dominance orientation focuses on an individual’s feelings about hierarchy and dominance in society more broadly.

#### 3.4.3. Perceptions of control

Perceptions of control were measured using three survey questions designed to capture perceptions of control over network positions and work, for example, “Our team had control over procedural decisions in the experiment.”

#### 3.4.4. Coordination

We measured coordination during the production period using Lewis’s (2003) subscale from the transactive memory systems measure. We used the coordination subscale to account for coordination benefits for groups that received their choice of central member. We performed an analysis to determine the reliability of the coordination subscale. The rwg(j) was 0.87, and Cronbach’s alpha was 0.84, within the acceptable range. The inter-class correlations were also in the acceptable range [ICC(1) = 0.37, ICC(2) = 0.60, *p* < 0.01].

### 3.5. Demographics

We captured demographic variables such as age, race, and gender.

## 4. Results

We present summary statistics and correlations in Tables 1, 2. Table 1 contains variables for the individual-level analysis around the selection of a central member, and Table 2 contains group-level variables to analyze performance.

**TABLE 1:** Summary statistics and pairwise correlations for individual-level variables.

	Mean	SD	Selected	Communication activity	App Inventor familiarity	Self-monitoring	Dominance motivation	Age	Male	Asian
Selected	0.333	0.473	–							
Communication activity	27.325	13.146	0.336***	–						
App Inventor familiarity	1.317	0.618	0.239***	0.001	–					
Self-monitoring	57.967	7.677	0.041	−0.005	0.068	–				
Dominance motivation	10.699	2.409	0.096	0.041	−0.007	0.020	–			
Age	21.699	3.011	−0.073	−0.016	−0.109	−0.099	0.095	–		
Male	0.618	0.488	0.095	−0.048	0.104	−0.148	0.048	0.156*	–	
Asian	0.650	0.479	−0.133	−0.059	−0.080	−0.204**	0.001	−0.034	−0.015	–

^+^*p* < 0.10, *p < 0.05, **p < 0.01, ***p < 0.001.

**TABLE 2:** Summary statistics and pairwise correlations for group-level variables.

	Mean	SD	Position chosen	Errors	Communication activity	App Inventor familiarity	Control	Coordination
Position chosen	0.439	0.502	–					
Errors	11.341	6.751	−0.384**	–				
Communication activity	1.902	10.452	0.440***	−0.439***	–			
App Inventor familiarity	0.1335	0.565	0.029	0.162	0.175	–		
Perceptions of control	11.447	2.077	0.278*	−0.054	0.061	−0.071	–	
Coordination	16.650	3.196	0.337*	−0.293*	0.252	−0.214	0.353**	–

^+^*p* < 0.10, *p < 0.05, **p < 0.01, ***p < 0.001.

We perform analyses at different levels to investigate the hypotheses. First, we investigate central member selection at the individual level to test Hypotheses 1a and 1b, as these hypotheses were about individual’s preferences. Next, we move to group-level analysis to examine the effects of central member selection on team performance to test Hypotheses 2, 3a, and 3b, as team performance is a group-level variable. We then perform robustness checks and investigate alternative explanations. We also perform a resampling procedure to compare position chosen with random assignment to explore whether we would have obtained findings consistent with our conclusion if we had used a different experimental design. Finally, we supplement our quantitative analysis with qualitative observations of group communication logs.

### 4.1. Member selection

To test Hypotheses 1a and 1b, we conducted analyses at the individual level, examining the factors that predicted selection to the central position. Members of all groups were asked for their choice of central member (regardless of condition), and these data collected after the group discussion regarding which member should occupy the central position allow us to capture group preferences in both conditions for who should occupy the central position, along with characteristics of the individual selected.

We performed a probit analysis at the individual level to determine individual characteristics that predicted selection to the central position. Each observation is a group member. Standard errors were clustered at the group level to account for within-group variance and interdependence. The results of the probit analysis are shown in Table 3. We enter all predictors separately and then enter predictors in one model in column 9 in Table 3.

**TABLE 3:** Probit results for individual selection to central position.

	(1)	(2)	(3)	(4)	(5)	(6)	(7)	(8)	(9)
Communication activity	0.035***							0.038***	0.038***
	(0.009)							(0.009)	(0.009)
App Inventor familiarity		0.396^+^						0.475*	0.430*
		(0.211)						(0.203)	(0.210)
Self-monitoring			0.007						0.007
			(0.017)						(0.018)
Dominance motivation				0.052					0.054
				(0.039)					(0.045)
Age					−0.032				−0.042
					(0.036)				(0.034)
Race (Asian)						−0.286			−0.252
						(0.232)			(0.276)
Male							0.257		0.317
							(0.261)		(0.283)
Constant	−1.434***	−0.962***	−0.841	−0.991*	0.270	−0.253	−0.593***	−2.134***	−2.245
	(0.291)	(0.283)	(0.979)	(0.424)	(0.783)	(0.141)	(0.168)	(0.442)	(1.444)
Observations	123	123	123	123	123	123	123	123	123

Standard errors in parentheses, clustered by group. ^+^*p* < 0.10, *p < 0.05, **p < 0.01, ***p < 0.001.

The dependent variable in these analyses was whether an individual was selected by their group to occupy the central position. The first variable entered is the number of messages sent by the focal individual during the training period (β = 0.04, *p* < 0.001). The more an individual communicated during the training period, the more likely they were to be selected as the central member, such that a one-standard deviation increase in communication yielded a 14% greater chance of selection to the central position. This result supports Hypothesis 1a and is shown in column 1 of Table 3.

Familiarity with App Inventor was a marginally significant predictor of selection to the central position (β = 0.39, *p* = 0.06). A one-standard deviation increase in relative App Inventor familiarity resulted in a 9% greater chance of selection. This effect is consistent with the idea that groups selected central members based on task expertise. This result provides some support for Hypothesis 1b and is shown in column 2 of Table 3. Results are consistent in column 8 of Table 3 when both communication and App Inventor familiarity are entered as predictors.

With respect to alternative predictors of selection, the analysis indicates that other variables (self-monitoring, dominance motivation, age, race, and gender) did not significantly predict selection (see columns 3–7 of Table 3). Only communication and App Inventor familiarity predicted selection to the central position. When all alternative predictor variables were included in the model, both communication and App Inventor familiarity remain significant (see column 9 of Table 3).

We also implemented a multi-level mixed effects probit with random slopes for group and found the same results. Communication (β = 0.04, *p* < 0.001) and App Inventor familiarity (β = 0.43, *p* = 0.03) predicted selection to the central position, with none of the other covariates predicting selection.

### 4.2. Explaining performance at the group level

To test Hypotheses 2 and 3, we move to the group-level and treat the team as the unit of analysis. Because of the importance of the central member in this network, however, we focus on the central member’s individual measures in communication and task expertise in our mediation analyses. We first determined whether receiving choice of central member had a significant effect on team performance. An independent samples *t*-test shows that it did, such that groups receiving their choice of central member made fewer errors (*M* = 8.44, SD = 6.92) than groups that did not receive their choice [*M* = 13.61, SD = 5.79; *t*(39) = 2.59, *p* = 0.013]. This represents a Cohen’s *d* of 0.81, a large effect size. This result supports Hypothesis 2.

To test Hypothesis 3a, we performed a mediation analysis to determine if communication activity explained the relationship between the manipulation and performance (Baron and Kenny, 1986). We acknowledge that communication activity was measured during the training period prior to the manipulation. However, it is essential to note that the manipulation was designed to induce a difference between the position assigned and position chosen conditions. This manipulation subsequently triggered a difference in the enduring characteristics of the central members. In light of this, we use the central members’ communication activity as a mediator.

First, consistent with the *t*-test results, the regression examining the relationship between the position chosen condition and errors shows that groups in the position chosen condition committed fewer errors than groups in the position assigned condition (β = −5.16, *p* = 0.02). Next, we regressed communication activity on the position chosen condition and found that the communication activity of the central member in the position chosen condition was marginally higher than in the position assigned condition (β = 6.86, *p* = 0.09). When we regressed performance on the manipulation and communication activity, the position chosen condition became marginally significant (β = −3.96, *p* = 0.06) and communication activity was negatively related to errors (β = −0.17, *p* = 0.02). These results (see columns 1, 2, and 3 of Table 4) suggest that choosing central members with higher communication activity mediates the negative effects of receiving one’s choice on errors. We also tested all mediation analyses with a bootstrapping procedure using the PROCESS macro for SPSS (Hayes, 2022). All analyses used 50,000 bootstrap percentile confidence intervals. We found a significant effect of the manipulation on performance, mediated by communication activity (95% CI: −4.85, −0.12). All the above analyses provide evidence for Hypothesis 3a.

**TABLE 4:** Ordinary least squares regressions for group performance.

	(1)	(2)	(3)	(4)	(5)
	Errors	Communication activity−central member	Errors	App Inventor familiarity−central member	Errors
Position chosen	−5.164*	6.865^+^	−3.965^+^	0.425*	−4.654*
	(2.028)	(4.049)	(2.069)	(0.181)	(2.209)
Communication activity (central member)			−0.175*		
			(0.0713)		
App Inventor familiarity (central member)					−1.201
					(1.943)
Constant	13.61***	25.91***	18.14***	1.130***	14.97***
	(1.212)	(2.773)	(2.117)	(0.0720)	(2.507)
Observations	41	41	41	41	41
*R* ^2^	0.148	0.068	0.257	0.142	0.156

Standard errors in parentheses. ^+^*p* < 0.10, *p < 0.05, **p < 0.01, ***p < 0.001.

We next tested Hypothesis 3b. Similar to the approach taken in Hypothesis 3a, we use the central member’s App Inventor familiarity, as measured after the training period, as a mediator. We found that App Inventor familiarity for the central member was higher in the position chosen condition (*M* = 1.56, SD = 0.71) than in the position assigned condition (*M* = 1.13, SD = 0.35), and this difference is statistically significant [*t*(38) = −2.44, *p* = 0.02].

We performed a mediation analysis to determine if relative App Inventor familiarity explained the relationship between the manipulation and performance (see columns 1, 4, and 5 of Table 4). First, as noted previously, groups in the position chosen condition committed fewer errors than groups in the position assigned condition (β = −5.16, *p* = 0.02). Second, we regressed App Inventor familiarity on the position chosen condition and found that App Inventor familiarity of the central member in the position chosen condition was higher than in the position assigned condition (β = 0.43, *p* = 0.02). Finally, we regressed performance on the manipulation and App Inventor familiarity. The position chosen variable decreased in significance and magnitude (β = −4.65, *p* = 0.04), whereas App Inventor familiarity was not significant (β = −1.20, *p* = 0.53). Using PROCESS (Hayes, 2022), we did not find that App Inventor familiarity mediated or explained the effect of the manipulation on performance (95% CI: −2.36, 1.23). Taken as a whole, this analysis does not provide evidence for Hypothesis 3b. We further discuss these results in the discussion section.

**FIGURE 2:**
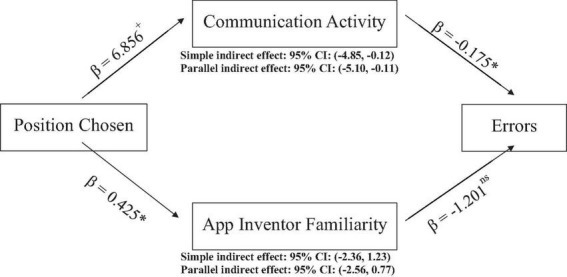
Simple and parallel mediation analyses, position manipulation, communication activity, App Inventor familiarity, errors. ^+^*p* < 0.10, *p < 0.05, ^**^p < 0.01, ^***^p < 0.001.

We supplemented our mediation analysis to test Hypotheses 3a and 3b with a parallel mediation analysis. Parallel mediation allows for a simultaneous test of whether both communication activity and App Inventor familiarity mediate the relationship between the manipulation and performance. We found, similar to above, that communication activity was a significant mediator (95% CI: −5.10, −0.11), but that App Inventor familiarity was not (95% CI: −2.56, 0.77). Figure 2 summarizes our mediation analyses, showing the simple indirect effects of each mediator and the parallel mediation effects.

### 4.3. Robustness checks and alternative explanations

As previously mentioned, one alternative explanation for Hypotheses 2 and 3 is that individuals receiving their choice of central member may be more motivated and perform better because they perceive control over their outcomes (Fisher, 1978; Spector, 1986). We investigated whether perceptions of control indeed differed between the two conditions. We found that perceptions of control were marginally higher in the position chosen (*M* = 12.09, SD = 1.45) than in the position assigned (*M* = 10.94, SD = 2.37) condition, [*t*(39) = −1.81, *p* = 0.08]. Although participants were told that all positions were randomly assigned, merely receiving their choice of central member induced somewhat greater feelings of control.

Next, we investigated whether perceptions of control mediated the relationship between position chosen and performance. When we regressed perceptions of control on position chosen, we found a marginally significant relationship, such that perceived control is higher when groups receive their choice (β = 1.15, *p* = 0.06). When we regressed performance on both position chosen and perceptions of control, we found a significant negative relationship between position chosen and performance (β = −5.38, *p* = 0.02) and an insignificant relationship between perceptions of control and performance (β = 0.19, *p* = 0.71). These results are shown in models 1, 4, and 5 of Table 5. On the whole, we did not find evidence that perceptions of control influenced performance. Furthermore, we included perceptions of control as a covariate and repeated the test of communication activity as a mediator of the effect of choice on performance. This mediation remained significant (95% CI: −5.07, −0.06). Thus, we found no evidence that including perceptions of control as a covariate altered our results.

**TABLE 5:** Robustness checks and alternative explanations.

	(1)	(2)	(3)	(4)	(5)
	Errors	Coordination	Errors	Perceptions of control	Errors
Position chosen	−5.164*	2.142*	−4.332^+^	1.151^+^	−5.379*
	(2.028)	(0.963)	(2.269)	(0.601)	(2.227)
Coordination			−0.388		
			(0.386)		
Perceptions of control					0.187
					(0.494)
Constant	13.61***	15.71***	19.71**	10.94***	11.56*
	(1.212)	(0.626)	(6.443)	(0.496)	(5.369)
Observations	41	41	41	41	41
*R* ^2^	0.148	0.113	0.178	0.077	0.151

Standard errors in parentheses. ^+^*p* < 0.10, *p < 0.05, **p < 0.01, ***p < 0.001.

We also examined whether coordination mediated the relationship between choice of central member and performance because groups receiving their choice of central member may benefit their coordination (see columns 1, 2, and 3 of Table 5). Coordination is higher when groups receive their choice of central member than when they do not (β = 2.14, *p* = 0.03). When performance is regressed on position chosen and coordination, position chosen is marginally significant (β = −4.33, *p* = 0.06) but coordination is not (β = −0.388, *p* = 0.321). Bootstrapping confirms that coordination does not mediate the relationship between the manipulation and performance (95% CI: −2.81, 0.92).

Finally, we captured logs of group discussions during the period in which group members were asked to discuss their choice of central member and analyzed these logs to illuminate the quantitative findings. First, we employed Linguistic and Word Count-22 (LIWC-22), computerized text analysis software that counts terms in a text to derive psychological assessments (Boyd et al., 2022). We focus on the emotional tone measure, which is derived from an algorithm that captures words indicating both positive and negative emotional tone. Higher scores on this measure indicate more positive emotional tone, with a score 50 representing a neutral tone.

We first examined whether teams experienced higher or lower positive emotions after being assigned their central member. We measured emotional tone for teams^1^ for the training period and for the post-manipulation period and calculated a difference score for each team. A positive difference score indicates an increase in positive emotion language after the manipulation and a negative score indicates the reverse. We find that teams in the position chosen condition experienced an increase in positive emotional tone after being assigned their central member (*M* = 4.479, SD = 23.248) and that teams in the position assigned condition experienced a decrease (*M* = −2.808, SD = 24.724). However, this difference was not statistically significant between conditions (*t* = −0.937, *p* = 0.355).

Next, we examined only the post-manipulation period during which performance was measured. We find that emotional tone was higher on average for teams in the position chosen condition (*M* = 48.473, SD = 14.514) than the position assigned condition (*M* = 37.833, SD = 17.203). This difference was statistically significant (*t* = −2.063, *p* = 0.046), indicating that groups who received their choice of central member communicated more positively than teams who did not receive their choice of central member. When we entered the emotional tone measure as a co-variate in a regression predicting performance, however, we found that emotional tone was insignificant (β = −0.062, *p* = 0.339) and our manipulation still had a negative and significant effect on errors (β = −4.768, *p* = 0.042). Lastly, we find that emotional tone did not mediate the relationship between the manipulation and performance (95% CI: −2.378, 1.050). Thus, though the tone of communication varied somewhat after making a choice, this did not explain the differences in group performance.

When viewed in combination with the results on perceptions of control, we find convergent evidence that although motivation–as reflected by perceived control over procedural decisions and emotional tone in communication–may have differed between conditions, motivation did not explain the effect of positions being chosen on performance. These analyses provide further evidence to suggest that the placement of the team’s preferred member in the central position indeed benefited performance and this benefit was not due to psychological benefits of receiving their choice but instead to the qualities of the member in the central position.

### 4.4. Supplemental analysis: synthetic random assignment

Our study compared groups that received their choice of central member to groups that did not receive their choice of central member. Our data also permit us to explore a different comparison, whether groups that receive their choice of central member perform better than groups in which the central member is randomly assigned. In the position assigned condition in our study, participants did not receive their choice of a central member. If we had used random assignment, however, groups would have received their choice of central member one-third of the time by chance, and two-thirds of the time, they would not receive their choice. Thus, a comparison of choice versus random assignment tests a different null hypothesis than our experimental design and allows us to determine if a different experimental design would have led to the same conclusions.

We constructed a dataset approximating random assignment by randomly drawing observations from both conditions. From the original data, we randomly sampled 6 observations from the position chosen groups and 12 observations from the position assigned groups. This yielded 18 observations in a synthetic random assignment condition where one-third received their choice of central member and two-thirds received a different central member. Recall that all groups were told that their central member had been randomly assigned so all were treated consistently.

We developed a bootstrapping procedure whereby we resampled from our original dataset to generate 50,000 sets of 18 synthetic random observations. We then compared each of the synthetic random datasets to groups in the position chosen condition in our original data and obtained test statistics and *p*-values.^2^ Recall that when we tested Hypothesis 2 and compared position chosen to position assigned, we saw a significant mean difference such that the position chosen group made about 5 fewer errors than the position assigned group (*p* = 0.013). For the synthetic random datasets, ninety-nine percent of the mean differences in performance between the original position chosen condition and the resampled synthetic random condition were in this same predicted direction, such that groups that received their choice of central member performed better than groups whose central member was randomly assigned. Twenty-one percent of the *p*-values were below the 0.05 threshold, and 39% of the *p*-values were below the 0.10 threshold. Thus, the difference between the position chosen condition and random assignment was almost always in the predicted direction and statistically significant some of the time.

### 4.5. Supplemental analysis: qualitative

In addition to the quantitative analysis of the chatlogs, we also read the logs in detail, and an investigation of the content revealed two themes. The first of these was communication activity: members discussed the importance of communication in making their choice. A participant who nominated another member wrote, “so just to confirm, you will take care of facilitating the communication between all of us.” Another participant, in discussing skills of other members, wrote, “[the other member] is really bad at communicating,” implying that a particular member should not be placed in the center position. Participants also recognized their own communication activity: “I feel like we communicated the best, so one of us should probably be in the middle though haha.”

The second theme was App Inventor familiarity. Participants recognized one another’s App Inventor experience by working together on the practice task. One participant nominated another groupmate because “he has a technical background.” Another participant stated, “i feel like u have a little more coding knowledge so you should be in the middle.” Some participants removed themselves from the running for the central position, noting that they had no programming experience: “I think i should be either 1 or 3…i have no knowledge about computing.”

## 5. Discussion

This study integrates the Carnegie perspective with the social psychology literature to show that allowing group members to choose who occupies which network positions enables teams to optimize their position assignments based on individuals’ skills and expertise. Guetzkow and Simon (1955) showed that groups in different network conditions were able to develop organizational arrangements that optimized their performance. We complement this work by showing that allowing teams to choose who occupies which network positions improves team performance. Team members are more likely to choose individuals who communicate frequently and those who appear to possess task expertise to occupy the central network position. For groups that received their choice of who occupies the central network position, choosing someone who communicates frequently explains their superior performance.

To test Hypotheses 1a and 1b, we conducted analysis at the individual level to determine the characteristics that predicted selection to the central network position. We found that both communication activity and App Inventor familiarity predicted selection to the central position, with communication activity being the more robust predictor. We conducted analysis at the group level to test Hypotheses 2, 3a, and 3b. We found that groups receiving their choice of central member performed better than groups not receiving their choice, providing evidence for Hypothesis 2, and that this effect is driven by the ability of groups receiving their choice to place members with high communication activity in the central network position, providing evidence for Hypothesis 3a but not for Hypothesis 3b.

We found that communication activity both predicted selection to the central position and mediated the relationship between choice and performance. This finding suggests that one reason why selection of network positions could improve performance is due to the ability to match member expertise to the requirements of the network position. When groups in this study received their choice of central member, this central member could transfer key task information, organize the work of the group, and delegate sub-tasks, leading to better team performance.

App Inventor familiarity predicted selection of group members to the central position, and thus App Inventor familiarity of the central member differed significantly between the two conditions. However, App Inventor familiarity did not mediate the relationship between the position manipulation and performance. Because the average App Inventor familiarity of the sample was low, it could be the case that, although members could identify when there were differences in ability, actual differences in ability were not sufficient to contribute to group performance. This finding suggests an interesting nuance to the expertise recognition literature. Identifying an expert member is only the first step for groups to benefit from their members’ expertise. For groups to tangibly benefit from members’ expertise, they must utilize the expert members’ skills, and these skills must be at a level high enough to solve the group’s task. Bonner and colleagues identified two conditions that can facilitate a group’s ability to recognize member expertise: (1) groups need information to judge members’ relative competences, and (2) tasks should allow group members to exhibit substantial variation in performance (e.g., Bonner, 2004; Bonner et al., 2007). Our study suggests that while these two conditions may be sufficient for groups to “identify” an expert member, in order for groups to benefit from having an expert member, the skill of this expert member should be sufficiently high.

Participants in our study effectively identified group members’ expertise while working on a relatively complex task for a short amount of time (i.e., 15 min). Research on expertise recognition has shown that groups initially focus on diffuse status cues and with experience learn to focus on task-related expertise (Bunderson, 2003). Masking diffuse status cues with computer-mediated communication might have enabled groups to focus their communication around task-relevant content, rather than being distracted by extraneous factors. Taken together, these studies suggest that the salience or availability of diffuse status cues could be an important moderator in how groups’ tenure affects their ability to recognize and utilize members’ expertise and that impeding the availability of these diffuse status cues could lead groups to focus on communicating task-relevant information, making it easier to identify each member’s expertise. Masking diffuse status cues can generate effects similar to the intervention by Bonner and Baumann (2012) which asked members to focus on knowledge that they already know; this enabled members to better judge other members’ expertise and facilitated expertise recognition.

We investigated factors other than expertise that might lead to one’s selection to the network’s central position. In addition to demographic factors like age, race, and gender, we investigated personality characteristics. Self-monitoring has been found to predict whether an individual occupies a brokerage position (Mehra et al., 2001; Sasovova et al., 2010), where an individual connects otherwise unconnected others. In this experiment, the central network position is analogous to a brokerage position, as the central member connects two unconnected alters. We found that an individual’s self-monitoring did not predict whether that individual was chosen to occupy the central position. We found similar results for dominance motivation, which assesses an individual’s propensity to dominate in social situations.

## 6. Limitations and future work

In our study, we chose to use positions that differed in their centrality. Centralization captures the extent to which communication ties are concentrated in only one or a few members (Freeman, 1978). Centralization is a dimension of networks that is often analyzed. We studied the most fundamental form of centralization−one member connected to two other members who are not connected to each other. This core form of centralization is the basis for several structural relationships, including bridging a structural hole (Burt, 2004) and spanning a boundary. Given the frequent occurrence of the structure we studied and its importance in different theories, understanding how members were chosen for the central position and the effect of those choices on the group’s performance seemed an important endeavor.

Thus, we intentionally chose to constrain participants to communicate dyadically within a centralized communication network. The dyadic structures we examine are at the core of other communication structures. However, dyadic communication, while prevalent, is just one way group members communicate. Broadcast communication, where all members can simultaneously send and receive messages (e.g., group chats and video conferencing), is also used. Though broadcast communication has the potential to enrich decision making by incorporating diverse viewpoints, it also complicates the process. For example, a high volume of ongoing discussions could distract group members, reducing the effectiveness of collective decision making (Diehl and Stroebe, 1987). Especially when managing external relationships, a single point of contact can reduce confusion and miscommunication compared to if multiple group members provide competing or incompatible advice. Our research suggests that group members gain benefits from giving the right person the right role, in our case a communication role. However, in conducting our study in this way, we were not able to speak to questions about broadcast communication, which could be examined in future work.

By informing participants in both experimental conditions that their positions were randomly assigned, we deceived participants about the true manipulation—whether they were given their choice of central member. We did not think that this deception would be harmful to participants. Following the experiment, we debriefed participants in both conditions about the manipulation and revealed to them that position assignment was not random. We chose this design to minimize the chance that the knowledge of receiving one’s choice would influence the results. If we had a design where participants knew whether they received their choice or not, the resulting motivational effect of receiving one’s choice could have potentially confounded our results. This would complicate our examination of how having a central member who fits well in the central position affects group performance. In essence, two factors would have been affected by the manipulation: explicitly knowing that they received choice and getting their chosen member with the requisite knowledge and skill in the central position. Telling participants that the member was randomly assigned reduced the potential differences between conditions and allowed us to be more confident that effects were due solely to having a member with requisite skill in the position, and not greater motivation of participants because they got their choice.

Despite its benefits, it is vital to consider the potential costs of using deception in experiments. Avoiding harm to participants is, of course, a central concern. In addition, deception may erode participants’ trust in experimenters and change their behaviors in subsequent experiments, and thereby negatively impact future data collection involving the same participant pool. For example, Jamison et al. (2008) found more inconsistent participant behavior in subsequent experiments after deception was employed concerning their partners’ identities (human vs. computer). However, on average, attrition rates were not affected by deception. At the same time, Rahwan et al. (2022) found that deceiving participants about the study’s purpose did not significantly alter their behavior. Thus, while the negative effect of deception may vary depending on its nature and the participant behaviors of interest, we nonetheless strongly recommend future researchers carefully weigh the implications of deception, consider norms about deception for their field, and thoroughly assess its necessity for their research questions.

Follow-on studies to our research can be done without deception. The current study provided evidence that being high in communication activity and having expertise in the technical aspects of the task led to a person being recommended for the central position. In the future, researchers could prescreen individuals on their communication activity and familiarity with the task and then randomly assign members high (or low) in these characteristics to the central position and assess the effect on performance. This design would allow for the researcher to determine the relative impact of member quality and position match on performance, though it could not answer the questions that our study did on group member preferences.

Finally, our findings contrast against purely structural perspectives suggesting that network structures lead to the same performance outcomes regardless of which positions individuals occupy. A well-established literature has argued that the structure of a group’s communication network influences performance and that these results are consistent within a given network structure (Shaw, 1964). In contrast, we show that group performance within a network structure is contingent on which individual group members occupy the network positions. The process by which individuals arrive at network positions has implications for group performance and advances recent interest in network formation (Ahuja et al., 2012) and psychology and social networks (Casciaro et al., 2015).

One boundary condition for our theory is that group members must have experience working together to accurately assess member skills for selection to network positions. If group members do not have experience working together, it could hinder their ability to identify members with skills appropriate for the network positions. For example, Yoon and Hollingshead (2010) found that in the absence of team communication, stereotyping was used to coordinate work across expertise areas. This inefficiency diminished and performance improved when communication was permitted. Whether this effect could be mitigated through knowledge repositories such as directories, LinkedIn, or personnel referrals is an interesting question for future research.

Additionally, we only investigated the effects of choice as it pertained to network positions in a single network structure. We did not investigate whether groups perform differently when they can choose their network structures, but we see this as a direction for future research. We expect that groups that can choose their network structure will select structures that fit the group’s skills, abilities, and preferred style of work. We also considered only teams that used computer-mediated communication. In teams where members work together face-to-face, additional factors may influence selection into network positions.

## 7. Conclusion

The Carnegie perspective saw organizational structures as deriving from the cognitive limits of individuals as information processors. We contribute to the Carnegie perspective by showing that the expertise of individual members also affects the development of organizational structures. More specifically, researchers in the Carnegie perspective analyzed how communication networks shape organizational structures and how those structures affect performance (e.g., Guetzkow and Simon, 1955). The Carnegie perspective, however, says little about the qualities of the individuals who occupy network positions—those who form the communication networks that enable work in organizations. As we illustrate, considering the network emergence process contributes to the Carnegie perspective and further, to the literature on social networks. We show that intra-team learning—where team members learn about one another’s skills—can facilitate the selection of appropriate members to occupy network positions and thereby improve team performance. When members choose who occupies central network positions, team performance improves. Choosing members who have the most expertise for the requirements of particular positions helps overcome the cognitive limitations of individuals.

A challenge in social network research is determining whether the results are due to the network’s structure or due to the processes through which the network was generated and the occupants of positions determined. Naturalistic studies of networks have been criticized for not accounting for the “endogeneity” of networks, that is, the process through which networks emerge (Manski, 1993). Although there have been calls for networks research to address endogeneity concerns (Ahuja et al., 2012), ours is the first experiment to compare the performance of networks in which members are assigned to a central network position with the performance of networks in which members receive their choice of central member. Our results indicate that allowing groups to endogenously choose who occupies the central position improves group performance. Attending to the endogenous selection process in future studies could help explain inconsistent findings in non-experimental studies. For example, Borgatti and Cross (2003) found that centralization harmed team performance, but Ehrlich and Cataldo (2014) found that network centrality facilitated performance. Our findings suggest that centralized groups in which members received their choices of member to occupy the central position are likely to perform better than groups where members do not choose position occupants. By taking into account endogenous member selection and position assignment processes, one arrives at a more accurate understanding of the effects of various networks (Manski, 1993; Gibson et al., 2021).

Our work also advances research on the recognition of expertise. Previous research had found that with experience working together, team members are able to identity each other’s expertise (Littlepage et al., 1997; Bonner, 2004) and further, that placing more weight on experts’ opinions improves team decisions (Bonner and Baumann, 2012). We extend the benefits of expertise recognition to choosing members for communication network positions and find that team performance improves when members with the requisite expertise are placed into appropriate positions. Thus, the recognition of expertise by team members provides a micro foundation for the more macro phenomena of communication network performance (Felin et al., 2015).

As our study demonstrates, the individuals occupying network positions and the process by which they arrive at those positions play a significant role in determining team performance. Structure can act as a constraint on how groups interact with one another, but the process of deciding who occupies which role in the structure is an important determinant of performance. The choices that drive the emergence of a network, when made with insight and information of the skills available in the team, help differentiate between good teams and exceptional ones.

## Data availability statement

The raw data supporting the conclusions of this article will be made available by the authors, without undue reservation.

## Ethics statement

The studies involving humans were approved by the Carnegie Mellon University Institutional Review Board. The studies were conducted in accordance with the local legislation and institutional requirements. The participants provided their written informed consent to participate in this study.

## Author contributions

JG, LA, and JK contributed to conception and design of the study. JG collected the data and performed the statistical analysis. All authors wrote sections of the manuscript, contributed to manuscript revision, read, and approved the submitted version.

## Footnotes

1 A chatlog for one team was not available and was excluded from the analysis.

2 We also performed a bootstrapping procedure and sampled, with replacement, from the original 18 observations that received their choice of central member. We drew 50,000 samples, obtained means, and compared differences with the synthetic random condition. The results did not substantively change.
